# Sliding-Window Normalization to Improve the Performance of Machine-Learning Models for Real-Time Motion Prediction Using Electromyography

**DOI:** 10.3390/s22135005

**Published:** 2022-07-02

**Authors:** Taichi Tanaka, Isao Nambu, Yoshiko Maruyama, Yasuhiro Wada

**Affiliations:** 1 Department of Science Technology of Innovation, Nagaoka University of Technology, Nagaoka 940-2188, Japan; 2 Department of Electrical, Electronics and Information Engineering, Nagaoka University of Technology, Nagaoka 940-2188, Japan; inambu@vos.nagaokaut.ac.jp (I.N.); ywada@vos.nagaokaut.ac.jp (Y.W.); 3 Department of Production Systems Engineering, National Institute of Technology, Hakodate College, Hakodate 042-8501, Japan; yoshiko@hakodate-ct.ac.jp

**Keywords:** electromyography, EMG, z-score, signal normalization, machine learning, classification model

## Abstract

Many researchers have used machine learning models to control artificial hands, walking aids, assistance suits, etc., using the biological signal of electromyography (EMG). The use of such devices requires high classification accuracy. One method for improving the classification performance of machine learning models is normalization, such as z-score. However, normalization is not used in most EMG-based motion prediction studies because of the need for calibration and fluctuation of reference value for calibration (cannot re-use). Therefore, in this study, we proposed a normalization method that combines sliding-window and z-score normalization that can be implemented in real-time processing without need for calibration. The effectiveness of this normalization method was confirmed by conducting a single-joint movement experiment of the elbow and predicting its rest, flexion, and extension movements from the EMG signal. The proposed method achieved 77.7% accuracy, an improvement of 21.5% compared to the non-normalization (56.2%). Furthermore, when using a model trained by other people’s data for application without calibration, the proposed method achieved 63.1% accuracy, an improvement of 8.8% compared to the z-score (54.4%). These results showed the effectiveness of the simple and easy-to-implement method, and that the classification performance of the machine learning model could be improved.

## 1. Introduction

Electromyography (EMG) is a biological signal whose amplitude fluctuates when exercising or contracting muscles. Many researchers have used this property to research and develop devices that are aimed at expanding and recovering human motor function [1,2,3,4,5]. Due to its easy design, which does not need a dynamics model and any physical parameters and only uses data, machine learning models have been used in many studies including motion control for artificial hands and gesture recognition using classifiers, and control of walking aids and assistance suits by predicting joint angles, joint angular velocities, or joint torque using regressors [2,3,4,5]. Linear models such as logistic regression and support-vector machines were first used around 2000, with an emphasis on improving classification performance by the feature extraction method such as mean absolute value, waveform length, and short-time Fourier transform [5,6,7,8]. However, as classification performance significantly improved with the development of deep learning [9] that occurred in 2012, research was also conducted to improve classification performance by changing the configuration of the deep neural network [10,11,12]. However, improving classification performance it is limited by the study of the feature and machine learning model alone. Therefore, methods other than feature-extraction and machine learning models are required to improve classification performance.

Data normalization is one of the methods for improving the classification performance of machine learning models and is used in fields such as imaging and biometrics [13,14,15]. Methods that are often used include min–max normalization [15,16], which normalizes the value range of the dataset from 0 to 1; and z-score, which normalizes the dataset mean to 0 and standard deviation to 1 [15,17]. Even in the field of EMG, the classification performance of models is improved by normalizing signals and features with z-score and min–max normalization [16,17,18,19,20]. Although the EMG fields use normalization such as maximum voluntary contraction (MVC) [21] or maximum voluntary isometric contraction (MVIC) [22] to enable motor analysis and motor performance evaluation between muscles and subjects, normalization is hardly used in EMG-based motion prediction research. Normalization is thought to be rarely used in motion prediction research for two possible reasons. The first reason is the need to measure the reference value (e.g., min, max, mean, or standard deviation of each EMG channel) to carry out the calibration. It can take 30 s–3 min to use the application, depending on the measurement method. The second reason is that reference values such as the max and mean of each EMG channel fluctuate that are due to various factors such as muscle fatigue, electrode position, and fluctuations in skin impedance [23,24,25,26,27]. Therefore, a reference value, once measured, cannot be re-used. This reduces the practical applications of normalization and makes it unsuitable for real-time processing (i.e., online processing). Therefore, we aimed to devise a normalization method that does not require calibration (i.e., measurement of reference values) and that is suited for real-time processing to enable normalization to be used as a means of improving the prediction performance of machine learning models.

We propose a normalization method that uses the sliding-window [28] and z-score normalization [15,17] shown in Section 2.1. The z-score is a simple normalization method that sets the dataset mean to 0 and standard deviation to 1. Compared to min-max normalization, which uses the minimum and maximum values in the entire dataset, the z-score uses the mean and standard deviation of the entire data, making it less susceptible to outliers. However, the z-score is usually not suitable for real-time processing because the entire dataset needs to be used for normalization, which incurs a time delay. Therefore, we considered combining the sliding-window analysis (SWA) that is used for signal analysis with time-varying parameter analysis. SWA involves analyses that use the signal within a specified window length. It is thought that using a signal of a sufficient length can achieve the same effect as the z-score that uses the entire dataset.

In recent years, research has focused toward enabling other people’s machine learning models to exhibit the same classification performance as machine learning models trained from their own data (i.e., improving generalizability) [29]. Studies that solve the problem of requiring individually specialized machine learning models by measuring a large amount of data for each user because of individual differences in myoelectric amplitudes have been reported. Methods have been proposed to reduce the required amount of own data by using other people’s data with domain adaptation, which technology enables the use of models that were trained in different datasets, even in datasets with different data attributes [29,30,31]. Such methods include geodesic flow kernel (GFK) [32], correlation alignment (CORAL) [33], and transfer component analysis (TCA) [34], which conducts motion prediction using a machine learning model trained from a different dataset after projecting one’s own data onto the data space, and domain adversarial neural networks (DANN) [35], which is a kind of deep learning method that trains the model to extract common features across different datasets.

The proposed method normalizes the standard deviation of myoelectric amplitude with individual differences, so it is thought that the influence of individual differences in myoelectric amplitude can be reduced, and the classification performance in the model learned from a different subject’s dataset can be improved. Compared to previous research such as DANN, the proposed method trains machine learning models using data other than one’s own data, so it is superior in that the models do not need to be trained for each user.

## 2. Methods

### 2.1. Proposed Sliding-Window Normalization

We propose a normalization method using sliding-window analysis (SWA) and z-score to improve the classification performance of the machine learning model and generalizability (i.e., exhibiting the same classification performance as the own machine learning model in the other’s machine learning model). SWA is used for signal analysis and time-varying parameter analysis using the signal within a specified window length [28]. SWA enables time series analysis by sliding the window so that when a new sample is obtained, the sliding window replaces the oldest sample with the new sample. The z-score is a kind of normalization method that is used to improve the classification performance of models in machine learning. The features are normalized by setting the feature mean to 0 and the standard deviation to 1 [15,17].

The proposed method is a combination of these two concepts and is called sliding-window normalization (SWN). As shown in Equation (1), the mean and standard deviation of the samples in the sliding window are set to 0 and 1, respectively.

(1) $$SWN\;EMG_{t,\;n-t+L_{\mathrm{norm}}}=\left(EMG_{n}-m_{t}\right)/s_{t}\;\;\left(t-L_{\mathrm{norm}}<n\leq t\right)$$

where *t* is the current discrete time, *L*_norm_ is the sliding window length, *n* is the discrete time number in the sliding window, *EMG_i_* is the *i*th processed EMG, *SWN EMG_t, n-t+L_*_norm_ is the myoelectric signal to which the *n-t+L*_norm_th proposed method (SWN) is applied at the *t*th, and *m_t_* and *s_t_* are the myoelectric mean and standard deviation on the *t*th sliding window, respectively. We used the “mean” and “std” functions in numpy in Python.

### 2.2. Comparison Methods

As comparison methods to SWN, applying z-score and none (without normalization).

#### 2.2.1. Z-Score

Z-score sets mean to 0 and standard deviation to 1 on a dataset [15,17]. Here, normalizing train and test dataset are based on train data like Equation (2).

(2) $$Z-Scored\;EMG_{t,\;\;d,s}=\left(EMG_{t,\;\;d,\;\;s}-\mu_{train,\;\;s}\right)/\sigma_{train,\;\;s}\;$$

where *t* is the current discrete time, *d* means the train data or test data, *s* is the subject number, *EMG_t,d,s_* is the *t*th processed EMG on *s*th subject, *Z-Scored EMG_t,d,s_* is the myoelectric signal to which *t*th z-score is applied at the *t*th processed EMG on *s*th subject, and $\mu_{train,\;s}$ and $\sigma_{train,\;s}$ are the myoelectric mean and standard deviation on *s*th subject’s training data.

#### 2.2.2. None (Without Normalization)

None apply nothing in the normalization process (Section 2.4).

### 2.3. Evaluation Method

This paper evaluates three types of items. The first is the improvement of the classification performance of machine learning models when the proposed method (SWN) is applied (Section 2.3.1), the second is the improvement of generalizability of machine learning models when the proposed method (SWN) is applied (Section 2.3.2), and the third is the improvement of the classification performance of the machine learning model when the number of subjects of the model that was trained with different data is increased by applying the proposed method (SWN) (Section 2.3.3).

Two types of machine learning models need to be trained. The first is the model trained with one’s own data (model type of OWN). The second is the model trained with another person’s data (model type of OTHER). Section 2.3.1 and Section 2.3.2 used models OWN and OTHER. Section 2.3.3 used only OTHER. Performance (OWN) involved dividing the data into training and testing datasets and calculating the performance using the model trained with one’s own training data and own test data. Performance (OTHER) involved calculating the performance using the model trained with another person’s training data and one’s own test data. The training data and test data was created by randomly dividing them into a 1:1 ratio every 10 consecutive trials.

#### 2.3.1. Normalization Evaluation

The evaluation of model classification performance improvement by the proposed method (SWN) was conducted by comparing the “classification performance in the model with SWN (OWN or OTHER)” and “classification performance of the model with z-score or None (OWN or OTHER)”.

Improvements in model classification performance that were due to the proposed model will be indicated by higher performance and lower standard deviation in performance. We consider the model classification performance improved and the research objective achieved when the performance (OWN or OTHER) with SWN applied is equal to or greater than the performance (OWN or OTHER) with z-score or None (no normalization).

#### 2.3.2. Generalizability Evaluation

The evaluation of generalizability was conducted by comparing the “classification performance in the model trained with one’s own data (OWN)” with the “classification performance in the model trained with another person’s data (OTHER)”.

Better generalizability is indicated by higher performance and lower standard deviation. Generalizability is considered improved and the research objective achieved when the performance (OTHER) with SWN applied is equal to or greater than the performance (OWN) without normalization (None) applied, and the performance (OTHER) with SWN applied is equal to or greater than the performance (OWN) with SWN applied.

#### 2.3.3. Evaluation on SWN Increased Number of Subject to Train Model

We investigated whether the classification performance of the model could be improved by increasing the number of subjects used for learning the model. We compared a model trained with nine subjects (OTHER) with a model trained with one subject (OTHER). The model trained with nine subjects (OTHER) was considered better if its performance was higher and its performance had a lower standard deviation.

#### 2.3.4. Evaluation Index

The accuracy shown in Equation (3) was used as the evaluation index for the classification performance of the machine learning model. Accuracy is an evaluation index that can simply compare results with multiple targets.

(3) $$\mathrm{Accuracy}=\frac{\mathrm{Success}\;\mathrm{Predictions}}{\mathrm{Success}\;\mathrm{Predictions}+\mathrm{Failure}\;\mathrm{Predictions}\;}$$

The Wilcoxon rank-sum test was used for significance tests. The significance level was set for the *p*-value less than 0.05. The “ranksums” function in scipy.states in Python was used for implementation. The “multipletests” function in statemodels.sandbox.stats.multicomp in Python was used for multiple comparisons. We used the Bonferroni correction as the correction method for the *p*-value.2.3.5. Machine Learning Model

We chose multi-class logistic regression for the machine learning model, which allows multi-class classification and short training time, to easily confirm the improvement by the proposed SWN. The “LogisticRegression” function in scikit-learn in Python was used for implementation. The parameters were as follows: penalty = “none”, class_weight = “balanced”, and max_iter = 6000. This model transforms the feature that is extracted from EMG (Session 2.4) to elbow-joint movement: rest, flexion, or extension (Session 2.6). The number of models trained was calculated by _number of subject_C_number of subject to train_.

### 2.4. EMG Processing

Before training the machine learning model, the measured EMG underwent preprocessing, normalization, feature extraction, and decimation.

Preprocessing involved the application of a low-pass Butterworth filter (3rd order, 500 Hz), decimation (2000 → 500 Hz), and a high-pass Butterworth filter (3rd order, 30 Hz). We used the scipy.signal “butter” function and “sosfilt” in Python for implementation. Normalization involved the application of either SWN, z-score, or no normalization (i.e., None). The window length for SWN was set at between 100 and 500 ms, with 100 ms intervals, because too long a window length decreases the amount of data. To adjust the amount of data, the data near the beginning of the trial are reduced based on the longest window length. In the case of “z-score and None”, the obtained features did not change even when the normalization window length was changed.

Feature extraction involved the calculation of the following six features to investigate window length for normalization and feature-extraction, for which high classification performance was obtained in previous studies: mean absolute value: MAV (Equation (4)) [6], mean waveform length: MWL (Equation (5)) [7], and difference root mean square: DRMS (Equation (6)) [7] as time-dimension features, short-time Fourier transform: STFT [5], and stationary wavelet transform: SWT [8] as frequency-dimension features, and combination of all five features: ALL. STFT involved averaging in the 1–70 Hz (low component), 60–100 Hz (middle component), and 100–250 Hz (high component) ranges and concatenating them (Equation (7)). SWT involved time-frequency conversion using Daubechies wavelet 2 (db2) as the mother wavelet and taking the absolute mean of the wavelet coefficient of level 3 frequency (cD3) as the feature.

(4) $$MAV_{t}=1/L_{\mathrm{feature}}\sum_{n=0}^{L_{\mathrm{feature}}-1}\left|EMG_{t-n}\right|$$

(5) $$MWL_{t}=\frac{1}{L_{\mathrm{feature}}-1}\sum_{n=0}^{L_{\mathrm{feature}}-2}\left|EMG_{t-n}-EMG_{t-n-1}\right|$$

(6) $$DRMS_{t}=\sqrt{\frac{1}{L_{\mathrm{feature}}-1}\sum_{n=0}^{L_{\mathrm{feature}}-2}{\left(EMG_{t-n}-EMG_{t-n-1}\right)}^{2}}$$

(7) $$STFT_{t}=cat\left(Low,Mid,Hig\right)\;Low=\frac{1}{bin_{\mathrm{low}}}\overset{70}{\underset{freq=1}{\sum }}{\mathrm{MeanSpec}}_{freq}\left(EMG_{\left(t-L+1\right)-t}\right)\;Mid=\frac{1}{bin_{\mathrm{mid}}}\overset{100}{\underset{freq=60}{\sum }}{\mathrm{MeanSpec}}_{freq}\left(EMG_{\left(t-L+1\right)-t}\right)\;Hig=\frac{1}{bin_{\mathrm{hig}}}\overset{250}{\underset{freq=100}{\sum }}{\mathrm{MeanSpec}}_{freq}\left(EMG_{\left(t-L+1\right)-t}\right)$$

where *t* is the current discrete time, L_feature_ is the window length of feature extraction, cat(·) is the concatenation function, *freq* is the frequency, MeanSpec is the function that outputs the spectrogram averaged in the time direction, and *bin* is the number of discrete frequencies in each of the low/middle/high frequencies. A Hanning window with a window length of 64 samples was used for the STFT window function. The functions in scipy.signal in Python were used for implementation. SWT is a method that improves the position invariance, which was a problem of wavelet transforms (WT), and the same mother wavelet as in WT can be used. The “swt” function in the pywt module in Python was used for implementation. The window length of feature extraction was set between 100 and 500 ms, with 100 ms intervals, because too long a window length decreases the amount of data.

Finally, decimation involved reducing the sampling rate of the features from 500 Hz to 20 Hz to reduce the amount of data and shorten the training time of the machine learning model.

### 2.5. Data Acquisition

#### 2.5.1. Subjects

The ethics board of the Nagaoka University of Technology approved this study according to the Declaration of Helsinki. The subjects were 10 right-handed 22- to 23-year-old men. The subjects were informed about the experiment in advance and consented to participate in the experiment.

#### 2.5.2. Experiment

**Figure 1:**
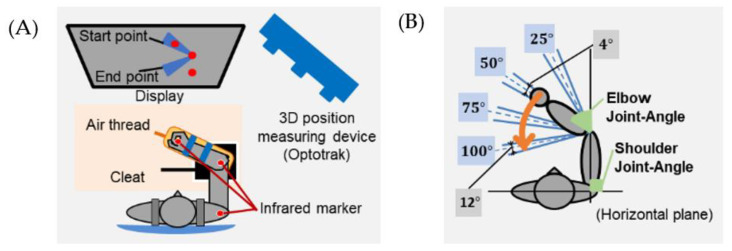
Experiment Condition: (**A**) state, (**B**) task.

The positions of the hands, elbows, and shoulders, and the EMG of the forearm and upper arm muscles, were measured as in the experimental environment shown in Figure 1A. Subjects performed 12 types of elbow single-joint movements with four different start points and end points as tasks (Figure 1B). A task involves moving from one of the four points (start point) to one of the other three points (end point). Each trial consisted of pre-rest (2 s), task (2.5 s), and post-rest (0.1 s); 36 trials (12 movements × 3) were conducted in one session, for a total of 10 sessions (i.e., 360 trials). The tasks were randomly selected for each session. The following four rules were also set as the success conditions for the tasks.

(1) No exercise during the rest period. Elbow joint angular velocity does not exceed 2 deg./s during the rest period.
(2) End the task during the task period. End the task between 0–2.5 s.
(3) Place the elbow joint angle at the start point (±2°.) during the rest period and at the end point (±6°.) at the end of the task.
(4) Place the shoulder and elbow joints within 3 cm of the initial position between the pre-rest and post-rest.

The position data were measured at three locations, namely, the hand, elbow, and shoulder, using Optotrack Certus, (NDI Inc., Waterloo, Canada, sampling rate: 500 Hz). The EMG was measured at the biceps brachii (×4), brachialis (×1), brachioradialis (×1), anconeus (×1), triceps brachii (outside) (×2), triceps brachii (long head) (×2), and extensor carpi radialis longus (×1), totaling 12 locations, by using Trigno Lab Avanti (Delsys, Natick, MA, USA, sampling rate: 2000 Hz)

### 2.6. Position Processing

Position processing consisted of noise reduction, work space → joint angle space conversion, elbow joint angular velocity conversion, coding, and decimation to obtain the target (rest, flexion, and extension of elbow joint movement) from the positions of the hands, elbows, and shoulders obtained in the subject experiment.

For noise reduction, we applied a zero-phase low-pass Butterworth filter (2nd order 20 Hz). The Python scipy.signal “butter” and “sosfiltfilt” functions were used.

Work space → joint angle space conversion involved the conversion of the positions of the hand, elbow, and shoulder to the elbow and shoulder joint angles using Equation (8).

(8) $$\{\begin{array}{c} \theta_{sld}=\mathrm{atan}2d\left(a,\;b\right)-\mathrm{atan}2d\left(\sqrt{a^{2}+b^{2}-c^{2}},\;c\right)\\ \theta_{elb}=\mathrm{atan}2d\left(\sqrt{a^{2}+b^{2}-c^{2}},c\right)+\mathrm{atan}2d\left(\sqrt{a^{2}+b^{2}-d^{2}},\;d\right) \end{array}\;\;a=y_{hand}-y_{sld}\;\;b=x_{hand}-x_{sld}\;\;c=\left(a^{2}+b^{2}+L_{sld}{}^{2}-L_{elb}{}^{2}\right)/2L_{sld}\;\;d=\left(a^{2}+b^{2}-L_{sld}{}^{2}+L_{elb}{}^{2}\right)/2L_{elb}\;$$

where atan2d(*y*, *x*) is the function that calculates the angle [deg.] from the two-dimensional coordinate position, $x_{hand}$ and $y_{hand}$ are the hand position [m], $x_{sld}$ and $y_{sld}$ are the shoulder position [m], and $L_{sld}$ and $L_{elb}$ are the upper arm and forearm length, respectively [m].

Elbow joint angular velocity conversion involved the conversion of the joint angle to the joint angular velocity using Equation (9).

(9) $${\dot{\theta }}_{elb,t}=\left(\theta_{elb,\;t+1}-\theta_{elb,\;t}\right)f_{s}\;$$

where ${\dot{\theta }}_{elb,t}$ is the elbow joint angular velocity at the discrete time *t*, and $f_{s}$ is the sampling frequency.

Coding involved the conversion of the elbow joint angular velocity to the target using Equation (10). This target was used as the teacher data for model training.

(10) $${\mathrm{target}}_{t}=\{\begin{array}{c} \mathrm{flexion}\;\left({\dot{\theta }}_{elb,t}\geq 2.0\;\left[\deg./s\right]\right)\\ \mathrm{extension}\;\left({\dot{\theta }}_{elb,t}\leq -2.0\;\left[\deg./s\right]\right)\\ \mathrm{rest}\;\left(\mathrm{otherwise}\right) \end{array}$$

## 3. Results

Prior to evaluating the classification performance (Section 3.2) and generalizability (Section 3.3) of the machine learning model by the proposed SWN method, we investigated the effects of window length for feature extraction and normalization (Section 3.1). Thereafter, we investigated the effect of the number of subjects used in model OTHER (Section 3.4). The chance level of accuracy in all results was 33.3% (3 classes: rest, flexion, and extension).

### 3.1. Effect of Window Length

In model OWN, we investigated the effect of changing window length for feature extraction and normalization on accuracy.

**Figure 2:**
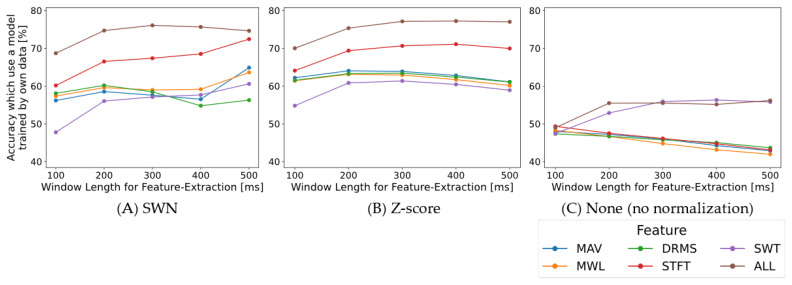
Effect of window length on feature-extraction (OWN). Window length for SWN is fixed at 500 ms. Horizontal axis indicates window-length for feature-extraction and vertical axis indicates accuracy using a model trained by data from own subject. Each color line shows results for the type of feature-extraction. (**A**) the results with the SWN (window length is fixed 500 ms), (**B**) the results with the z-score, and (**C**) the results with None (no normalization).

First, the effect of window length for feature extraction on accuracy was investigated. Figure 2 shows the results of changing the window length for feature extraction between 100 and 500 ms in 100 ms intervals and comparing the proposed SWN (the window length fixed at 500 ms), z-score, and None (no normalization) for the six types of features. Figure 2A shows that applying SWN improved accuracy as the window length for feature extraction increased. In contrast, with z-score and no normalization, the accuracy decreased as the window length for feature extraction increased (Figure 2B,C). We surmise that applying SWN improves the classification performance of the model by lengthening the feature extraction window.

**Figure 3:**
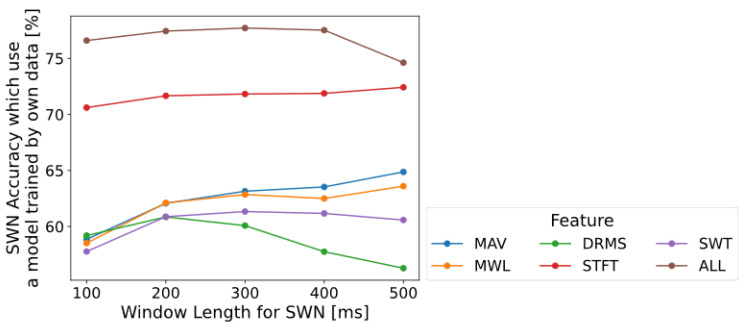
Effect of the window length for normalization (OWN). The window length for feature-extraction is fixed at 500 ms. Horizontal axis indicates window-length for normalization and vertical axis indicates accuracy using a model trained by data from own subject. Each color line shows results for the type of feature-extraction.

Next, the effect of window length for normalization on accuracy was investigated. We changed the window length for normalization between 100 and 500 ms at 100 ms intervals, and the window length for feature extraction was fixed at 500 ms. Figure 3 shows the results of calculating with all six feature types. The accuracy fundamentally increases with the window length for normalization. We recommend that the window length for normalization should be selected within the range of 200–500 ms, with the window length that maximizes accuracy being selected.

We also investigated whether there was any synergy between normalization and feature extraction window length, but no synergistic effects were observed. This result is shown in Appendix A.

### 3.2. Comparison of Normalization Methods

We investigated whether the proposed method (SWN) would improve the classification performance of the models using all the features (ALL). Research in recent years has been conducted to reduce the pre-data measurement of each user by enabling others’ machine learning models to exhibit the same classification performance as one’s own model (i.e., improving generalizability). Therefore, in this study, we compared the accuracy of SWN, z-score, and non-normalization (None) between a model learned from one’s own data (OWN) and a model learned from other subjects’ data (OTHER). The window lengths for normalization and feature extraction were changed between 100 and 500 ms in 100 ms intervals, and the maximum accuracy was compared. The number of subjects used when training model OTHER was set to nine people.

**Figure 4:**
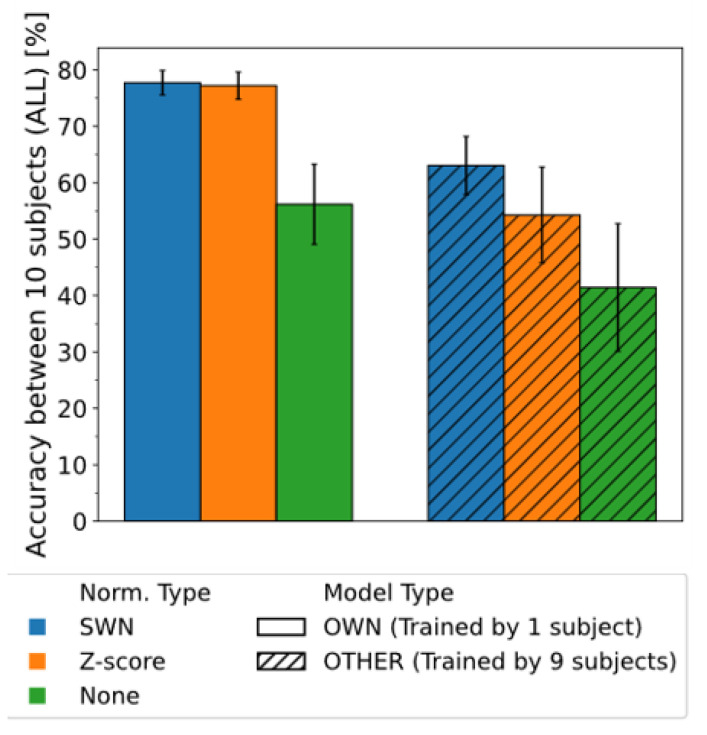
Performance comparison between normalization methods and model types using feature ALL. Horizontal axis indicates normalization methods and model types, and vertical axis indicates accuracy using a model trained by data from other or own subjects using feature ALL. SWN is the result of applying proposed normalization, z-score is the result of applying compared normalization and None is the result of not applying normalization.

Figure 4 shows a result comparing the accuracy of the model (OWN or OTHER) with SWN, z-score, and no normalization (None). A comparison between SWN_OWN (accuracy: 77.7 ± 2.9%, blue bar) and None_OWN (accuracy: 56.2 ± 7.1%, orange bar) shows that the mean accuracy of SWN_OWN significantly increased by 21.5% (Wilcoxon rank-sum test, *p* < 0.001) and its standard deviation of accuracy decreased by 4.9%. A comparison between SWN_OWN (accuracy: 77.7 ± 2.9%, blue bar) and z-score_OWN (accuracy: 77.2 ± 2.4%, green bar) shows that SWN demonstrated the same performance as z-score on the model type OWN (*p* > 0.05). These results show that the proposed SWN can improve the accuracy of machine learning model, much like the z-score when using the machine learning model that was trained from one’s own data. Furthermore, a comparison between SWN_OTHER (accuracy: 63.1 ± 5.1%, blue shaded bar) and None_OTHER (accuracy: 41.4 ± 11.3%, green shaded bar) shows that the accuracy of SWN_OTHER significantly increased by 21.6% (*p* < 0.01) and its standard deviation of accuracy decreased by 6.2%. A comparison between SWN_OTHER (accuracy: 63.1 ± 5.1%, blue shaded bar) and z-score_OTHER (accuracy: 54.4 ± 8.5%, orange shaded bar) shows that the accuracy of SWN_OTHER significantly increased by 8.8% (*p* < 0.01) and its standard deviation of accuracy decreased by 3.4%. These results show that the proposed SWN can improve the accuracy compared to the z-score when using other’s machine learning models. These two results show the effectiveness of the proposed method.

### 3.3. Generalizability Comparison

We investigated whether the proposed SWN method would improve generalizability (i.e., other’s machine model would exhibit the same classification performance as one’s own model). A comparison was made between the accuracy of model OTHER with SWN applied that was used in Section 3.2 in Figure 4 and model OTHER where normalization was not applied.

From Figure 4, a comparison between SWN_OTHER (accuracy: 63.1 ± 5.1%, blue shaded bar) and None_OWN (accuracy: 56.2 ± 7.1%, green bar) shows that SWN_OTHER had an accuracy that was 6.9% higher (*p* < 0.05) and standard deviation of accuracy that was 2% lower. However, a comparison between SWN_OWN (accuracy: 77.7 ± 2.2%, blue bar) and SWN_OTHER (accuracy: 63.1 ± 5.1%, blue shaded bar) shows that SWN_OWN had an accuracy that was 14.7% higher (*p* < 0.001) and standard deviation of accuracy that was 3% lower. These results show that the classification performance of the machine learning model was improved by the proposed SWN, but even a model that used a large amount of other’s data did not improve generalizability to the extent that it was similar to the classification performance using one’s own data.

### 3.4. Number of Subjects to Train Model (OTHER)

**Figure 5:**
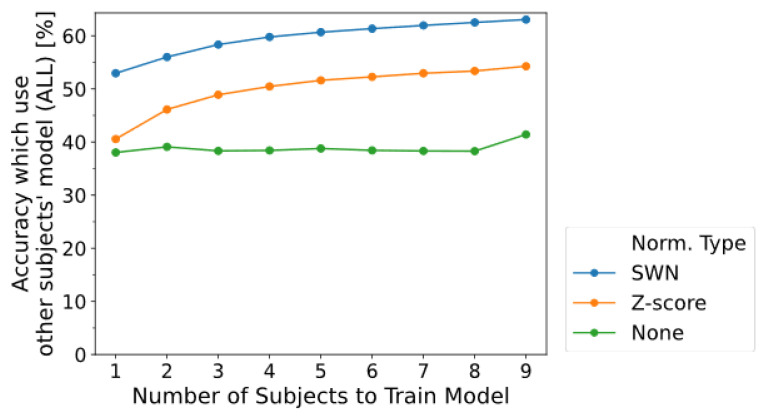
Effect of the number of subjects to train model (OTHER). Horizontal axis indicates number of subjects to train model (OTHER) and vertical axis indicates accuracy using a model trained by data from other subjects. Each color line shows results for the type of normalization.

It was shown in Section 3.2 that applying the proposed SWN method could improve the classification performance of not only the model trained from one’s own data (OWN) but also the model trained from other user’s data (OTHER). Therefore, investigating the extent to which the classification performance of the model (OTHER) could be improved by training the model by mixing the data of multiple other subjects. The number of subjects used for training the model was changed from 1 to 9. The window lengths for normalization and feature extraction were changed in the range of 100 to 500 ms in 100 ms intervals, and the maximum accuracy was compared. All the features (ALL) were used for the classification. From Figure 5, accuracy for feature ALL for cases of proposed SWN and z-score increased with subjects used in model training. In contrast, the accuracy did not either monotonically increase or decrease with respect to the number of subjects for cases without normalization (None). This implies that high classification performance can be achieved with an increase in the number of subjects by applying proposed SWN in cases that use others’ data.

Next, to investigate whether the increase in the number of subjects had a significant effect, we compared cases with either nine subjects (highest accuracy in Figure 5) and one subject (lowest accuracy in Figure 5) used in the training of the machine learning model. The results show *p* < 0.01 on proposed SWN, *p* < 0.001 on z-score, and *p* ≥ 0.05 on None (no normalization). It implies increasing the accuracy by normalization (proposed SWN and z-score).

## 4. Discussion

In this study, we proposed a new normalization method, SWN, to improve the classification performance of machine learning models. We succeeded in increasing classification accuracy from 56.2% to 77.7%, an increase of 21.5%, by applying the SWN (blue and green bar with no line in Figure 4). Furthermore, the standard deviation of accuracy decreased from 7.1% to 2.9%, a decrease of 4.9%. The results show the effectiveness of the proposed method.

In this section, we discuss the performance of SWN compared with z-score and no normalization (Section 4.1), the parameters and features selection on SWN (Section 4.2), the factors that improve the model classification performance by the proposed method (Section 4.3), and the feasibility of real-time prediction (Section 4.4).

### 4.1. Performance of SWN

The proposed SWN can improve a classification accuracy (OWN) because the proposed SWN (77.7%) has a 21.5% higher accuracy than no normalization (56.2%) from Figure 4 in Section 3.2. However, the proposed SWN has the same accuracy as the z-score (77.2%) and is not better than the z-score. The advantage of the proposed SWN is that it normalizes EMG signals in each sliding window and does not need a reference value (e.g., min, max, mean, or standard deviation of each EMG channel). However, the z-score method normalizes the signal using all the data. If the measurement is done across days, the EMG signals may vary between days and the normalization could negatively affect the accuracy. The same effects are likely to occur in the case when the sensor placement changes and muscles fatigue. Therefore, we need to investigate whether the proposed SWN is better than the z-score using data that changes depending on the measurement day, sensor location, and muscle fatigue statement.

In recent years, research has focused on enabling other people’s machine learning models (model type of OTHER) to exhibit the same classification performance as machine learning models trained from their own data (model type of OWN). Therefore, we investigated whether the SWN proposed in Section 3.3 could deliver the same or higher performance than the model trained on our own data. As a result, SWN_OTHER (63.1%) had a 14.7% lower accuracy than SWN_OWN (77.7%); however, it was higher than z-score_OTHER (54.4%) and None_OTHER (41.4%). The proposed SWN has better model accuracy when using other people’s data than the z-score. This could be because SWN can normalize the myoelectric signal within the sliding window and the difference of data between subjects are reduced while z-score and None are influenced by such differences. This point is the advantage of the proposed SWN compared with the z-score. Furthermore, the accuracy increased with the number of subjects to train model (OTHER), much like the previous study [31,36,37]. Therefore, the proposed SWN has the same effect as the previous study’s methods. However, similar to previous studies, a large amount of subjects’ data is needed to obtain high classification accuracy when applying the proposed SWN to model OTHER.

### 4.2. Parameters and Features Selection of SWN

The parameters of SWN are the window length for normalization and feature extraction. They should be fundamentally set to long to improve the accuracy of the model when applying the proposed SWN. The window length for normalization should be set between 200–500 ms and the window length for feature extraction should be set at 500 ms from Figure 2 and Figure 3. Furthermore, the effect of window length was investigated by using data with a short trial of 4 s in this paper. However, if the data length is more than 4 s, increasing the window length for normalization and feature extraction to more than 500 ms may improve the accuracy of the model. Therefore, we need to investigate the effect of the window length on normalization and feature extraction for data, where one trial of the measurement experiment is longer than 10 s.

The feature STFT has the highest accuracy on SWN from the five feature types: MAV, MWL, DRMS, STFT, and SWT, as shown Figure 2. Although SWT has the highest accuracy, the other four features have almost the same accuracy on no normalization (None). Thus, even though high accuracy was obtained in the previous study, it may not be possible to obtain it in the case of the proposed SWN. Additionally, using multiple features (feature ALL) has a higher accuracy than single features (MAV, MWL, etc.) as shown in Figure 2 and Figure 3. Hence, classification accuracy can be enhanced by incorporating multiple features.

### 4.3. Analysis of SWN

**Figure 6:**
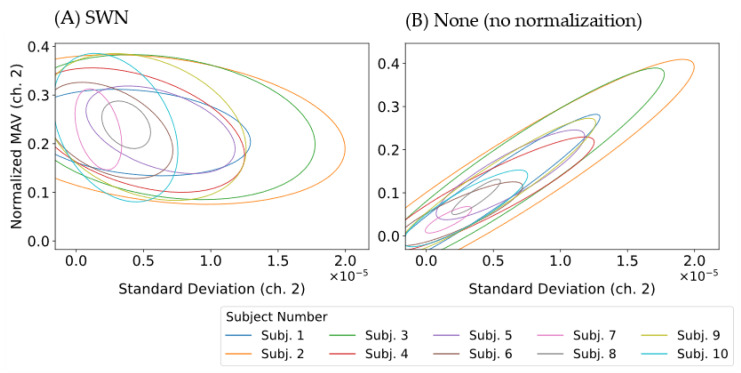
Example of the distribution analysis between standard deviation of EMG and feature each subject on the channel 2 (MAV). (**A**) Normalization (SWN), (**B**) No Normalization (None). Both MAV of SWN and None are normalized (max: 1.0) by maximum data in the 10 subjects.

We investigated the effect of dividing with the standard deviation of EMG, which was thought to have led to the improvement of the classification performance of machine learning models and is a feature of SWN. Visualizing the relationship of standard deviation of EMG and the feature of EMG by drawing a confidence ellipse with a standard deviation of 2. The “confidence_ellipse” function of matplotlib in Python was used for implementation. Figure 6 shows an example of the results of treating MAV as a representative of the features. The S.D. of EMG-MAV distribution in the case with normalization (SWN) had a weakly negative or no correlation, whereas the distribution in the case without normalization (None) had a strongly positive correlation.

The results obtained in Figure 6 are used as a basis for conducting a correlation analysis of cases with normalization (SWN) and without normalization (None). The representative feature was MAV, which was the same as in Figure 6. We calculated the correlation coefficient of the S.D. of EMG vs. MAV for each channel and subject, taking the mean value. The correlation coefficient was −0.33 on the with normalization (SWN) and 0.90 on the without normalization (None). Therefore, the S.D. of EMG and MAV had a weakly negative correlation for cases with normalization (SWN) and a strongly positive correlation for cases without normalization (None). These results imply that one of the factors that improved the classification performance of the machine learning model was the reduction of the influence of the standard deviation on the features by the proposed SWN method. 

### 4.4. Comparison of Calculation Time

SWN was effective in improving the classification performance of the machine learning model. However, it is still unknown whether this can satisfy the required execution speed for real-time processing. Therefore, the preprocessing and normalization shown in Section 2.4 were executed at intervals of 20 ms (50 Hz), and the mean execution time was compared between cases with normalization (SWN) and without normalization (None). The execution environment was as follows: Intel(R)Core (TM) i7-9700K CPU @ 3.60 GHz, Python 3.8.12. The result shows 409 µs (SWN) and 333 µs (None). The normalization rate in 409 µs was 18.6%, a minimal effect. It implies the proposed SWN can be implemented on a low-computing-power device such as a microcomputer. These results indicate that the proposed SWN method can be implemented in real time.

## 5. Conclusions

In this paper, we proposed a normalization method (SWN) that used the sliding window and z-score to improve the classification performance of devices using EMG. Applying SWN improved the accuracy by 21.5% compared to the case without normalization. Even when a machine learning model that was trained with other’s data was used, the accuracy improved by 21.6% compared to the case without normalization and 8.8% compared to the case with z-score. These results show that the classification performance of the machine learning model could be improved by the proposed method (SWN). Results of investigating the relationship between the standard deviation and features also show that applying the SWN changed the correlation between the standard deviation and features from that of a strongly positive one to a weakly negative one. This was assumed to be one of the factors that improved the classification performance of machine learning models.

The focus of future studies will be on the following two points. First, we found that the proposed SWN has a higher accuracy than the z-score on the model using other people’s data. However, the proposed SWN has almost the same accuracy as the z-score in the case of using own data. To determine whether the proposed SWN is superior to the z-score, we need to analyze in detail whether it is robust to various data attributes such as measurement day, sensor location, and muscle fatigue. Second, we applied the proposed SWN to the classification model. We need to investigate whether the proposed SWN can improve the performance of the regression model that predicts kinematic parameters such as the joint angle and angular velocity.

## Figures and Tables

**Figure A1:**
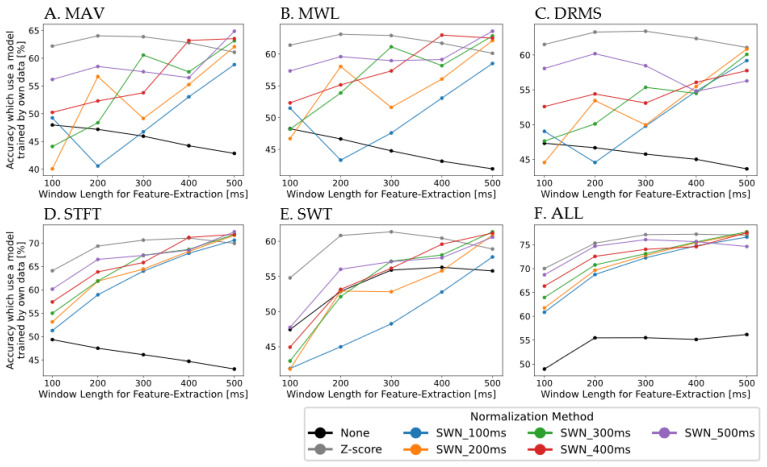
Effect of the window length for feature-extraction and normalization (OWN). Horizontal axis indicates window-length for feature-extraction and vertical axis indicates accuracy using a model trained by data from own subject. Each color line shows the normalization methods. The black line shows no-normalization (None), gray line shows z-score and other color line shows with normalization each window-length for normalization. (**A**–**F**) indicate feature-extraction methods.

## Data Availability

Not applicable.

## Appendix A. Effect of Window Length for Feature-Extraction and Normalization

In Section 3.1, we investigated the effect of window length by fixing the window length for feature extraction or normalization at 500 ms. In this session, we investigate the effect of window length by not fixing the window length for feature extraction and normalization. The window length for feature extraction and normalization is changed in the range of 100–500 ms in 100 ms increments. We compare proposed SWN, z-score, and None (no normalization) in the six types of features by using model OWN (Figure A1).

From Figure A1, the accuracy increases with window length for feature extraction, and does not significantly change with window length for normalization, like in Section 3.1. Furthermore, there are no interactions with window length for feature extraction and normalization.
